# Identification of *Leptotrichia hofstadii* as a Post-Treatment Recurrence Biomarker in Severe Early Childhood Caries

**DOI:** 10.3390/microorganisms14071513

**Published:** 2026-07-11

**Authors:** Yuchen Yin, Bingqian Zhao, Ruidi Li, Runkai Wang, Jiahan Peng, Bin Xia, Jing Tian

**Affiliations:** Department of Pediatric Dentistry, Peking University School and Hospital of Stomatology, National Center of Stomatology/National Clinical Research Center for Oral Diseases/National Engineering Research Center of Oral Biomaterials and Digital Medical Devices, Beijing 100081, China; 1810303123@pku.edu.cn (Y.Y.); zhaobingqian@pku.edu.cn (B.Z.); 1910303107@pku.edu.cn (R.L.);

**Keywords:** S-ECC, caries recurrence, *Leptotrichia hofstadii*, *Streptococcus mutans*, synergistic interaction, biofilm, dual-species transcriptomics

## Abstract

Recurrence remains a significant challenge following the treatment of Severe Early Childhood Caries (S-ECC). This study aimed to identify candidate recurrence-related biomarkers for S-ECC and elucidate their potential pathogenic mechanisms. Through metagenomic sequencing of supragingival plaque from 32 children at one month post-treatment, we identified *Leptotrichia hofstadii* as one of the potential biomarkers for S-ECC recurrence (AUC = 0.8438 for the sequencing set and AUC = 0.75 for the validation set). In vitro dual-species biofilm assays using crystal violet staining and Confocal Laser Scanning Microscopy (CLSM) demonstrated that *L. hofstadii* promotes early-stage *S. mutans* colonization and extracellular polysaccharide (EPS) formation through contact-dependent synergistic interactions. Scanning electron microscopy revealed that *L. hofstadii* may function as a spatial scaffold within dual-species biofilm. Furthermore, this synergy significantly accelerates environmental acidification, leading to earlier attainment of the critical demineralization threshold (pH 5.5). At the transcriptional level, carbohydrate metabolism-related pathways were upregulated in dual-species biofilm, including starch and sucrose metabolism, PTS and ABC transporters. Additionally, the *fruA* gene, which degrades fructan in EPS was downregulated in the dual-species biofilm compared with *S. mutans* monoculture. These findings suggest that *L. hofstadii* facilitates a cariogenic microenvironment by enhancing the metabolic activity of *S. mutans* biofilms. Collectively, this study identifies *L. hofstadii* as a potential biomarker for S-ECC recurrence prediction and provides preliminary insights into possible interspecies mechanisms, offering valuable clues for future research into targeted preventive strategies.

## 1. Introduction

Severe early childhood caries (S-ECC) remains a formidable global public health challenge, profoundly impacting the physical development and psychosocial well-being of young children. While the global prevalence of dental caries in the primary dentition is already substantial at approximately 43% [1], this burden is even more pronounced in China, where the prevalence of S-ECC exceeds 50% among preschool children [2]. Owing to limited patient cooperation at a young age, comprehensive dental rehabilitation under general anesthesia is frequently required. However, such intervention does not always result in stable long-term outcomes. Post-treatment recurrence rates remain alarmingly high, ranging from 24% to 59% within 12–24 months post-surgery [3,4,5]. In some cases, children even require secondary surgical intervention [6], imposing a heavy socioeconomic burden on families and healthcare systems.

Conventional predictive models for recurrence rely predominantly on clinical indicators, which lack the sensitivity and specificity of microbiological biomarkers required for early-stage risk assessment [7]. Prior research has established that the oral microbiota undergoes significant compositional shifts one month following comprehensive treatment [8]. The immediate post-operative period constitutes a decisive “ecological window” for identifying predictive microbial signatures [9]. Notably, the combined microbial indicators from the saliva microbiome—comprising genera such as *Fusobacterium*, *Prevotella*, *Leptotrichia*, and *Capnocytophaga*—have shown predictive utility for recurrence [10]. However, other investigations have identified associations between *Streptococcus parasanguinis*, *S. mutans* Cbp mutants, and *Candida albicans* with caries recurrence [11]. Collectively, these findings imply that the post-therapeutic oral environment in recurrent individuals may harbor a distinct microbial composition, suggesting that the ecological re-establishment in these subjects might follow a different trajectory from that of those not prone to recurrence. Previous studies predominantly utilized 16S rRNA gene sequencing. This methodology limits taxonomic resolution to the genus level and precludes high-resolution functional analysis at the species level.

As the predominant cariogenic pathogen, *Streptococcus mutans* plays an important role in cariogenesis through key virulence factors, including bacterial adhesion, acid production, acid tolerance, and extracellular polysaccharide (EPS) synthesis [12,13]. Investigating the interplay between *S. mutans* and potential recurrence biomarkers is essential to understanding the mechanisms of caries recurrence post-treatment.

Accordingly, our study employed metagenomic sequencing to identify recurrence-associated microbial markers at one month post-surgery. Furthermore, we investigated the synergistic cariogenic mechanisms between *L. hofstadii* and *S. mutans* through in vitro co-culture experiments, providing a foundation for novel, biofilm-targeted caries prevention approaches.

## 2. Materials and Methods

### 2.1. Study Cohort Design and Sequencing

#### 2.1.1. Subjects and Design

The study protocol was reviewed and approved by the Ethics Committee of the Peking University School and Hospital of Stomatology (PKUSSIRB-202056091). Prior to enrollment, written informed consent was obtained from the parents or legal guardians of all participants. All clinical procedures were conducted in strict accordance with the Declaration of Helsinki and relevant institutional guidelines. The study cohort initially comprised 69 children, aged 3 to 5 years, diagnosed with S-ECC and undergoing comprehensive caries treatment under general anesthesia from January 2021 to December 2022. Clinical diagnosis and caries assessment were performed using the International Caries Detection and Assessment System (ICDAS) and the American Academy of Pediatric Dentistry (AAPD) guidelines. Eligible subjects were required to have primary dentition with at least 18 teeth. Exclusion criteria included the presence of systemic diseases, a history of previous oral treatment, or visually distinguishable enamel/dentin hypoplasia. Additionally, participants were required not to have used antibiotics within the preceding two weeks, fluoride products within one month, or mouthwash on a long-term basis.

**Figure 1:**
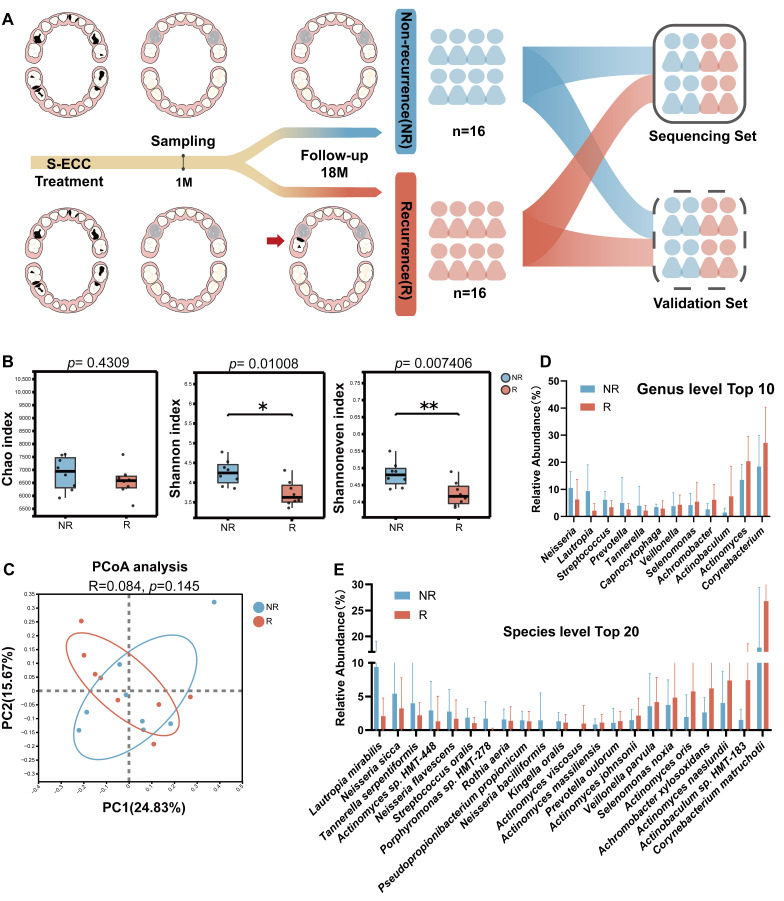
Study design and microbial diversity analysis of supragingival plaque samples collected at one month post-treatment (n = 8 per group). (**A**) Schematic illustration of the study design. Supragingival plaque samples were collected at one month post-operatively for metagenomic sequencing. The red arrow indicates the site of caries recurrence. (**B**) Alpha diversity comparison between non-recurrence (n = 8) and recurrence (n = 8) groups. (**C**) Principal coordinate analysis (PCoA) based on Bray–Curtis distance, illustrating the Beta diversity. (**D**) Relative abundance of the top 10 genera in non-recurrence (n = 8) and recurrence groups (n = 8). (**E**) Relative abundance of the top 20 species in non-recurrence (n = 8) and recurrence groups (n = 8). No significant differences were observed for any of these genera or species by Wilcoxon rank-sum test. * *p*-value < 0.05, ** *p*-value < 0.01.

Participants were monitored through an 18-month longitudinal observation period. Based on clinical outcomes, children who developed secondary, recurrent, or new caries were assigned to the recurrence group. Conversely, those whose primary teeth and restorations remained intact throughout the 18-month follow-up were included in the non-recurrence group. To ensure sufficient susceptible tooth surfaces for assessment, subjects with more than six preformed crowns were excluded. Ultimately, 16 cases were assigned to the non-recurrence group, and 16 cases were selected for the recurrence group, matched for age and dmft (decayed, missing, or filled teeth) indices. This study utilized a two-stage design with a 1:1 distribution, in which a sequencing set and a validation set (n = 8 per group each) were established based on enrollment order and statistical power considerations (Figure 1A).

Supragingival plaque was collected one month post-treatment from intact enamel using a sterile spoon excavator and transferred into 500 μL TE buffer (10 mM Tris–HCl, 1 mM EDTA, pH 8.0; Thermo Fisher Scientific, Waltham, MA, USA). Sampling was performed on the surfaces of teeth with intact natural enamel, avoiding surfaces with resin-based restorations, stainless steel crowns, sealants, or active carious lesions. To ensure maximum recovery of microbial biomass, the samples were subjected to thorough elution via vortexing for 60 s. Samples were transferred to −80 °C within two hours to maintain DNA integrity [8].

#### 2.1.2. Metagenomic Sequencing

Microbial DNA extraction and metagenomic shotgun sequencing were performed by Majorbio Bio-pharm Technology Co., Ltd. (Shanghai, China). Following DNA extraction and library construction, individual libraries were pooled based on effective concentration and target data volume requirements for sequencing on the Illumina HiSeq platform (San Diego, CA, USA). Taxonomic profiling was performed by aligning metagenomic reads against the MetaPhlAn 3.0 clade-specific marker gene database.

#### 2.1.3. Bioinformatics and Statistical Analysis

Ecological richness and evenness were assessed by Alpha diversity (Chao, Shannon, and Shannoneven indices). Beta diversity was visualized via Principal Coordinate Analysis (PCoA), with community structure differences determined through ANOSIM and PERMANOVA.

Differential taxa were identified using the Wilcoxon rank-sum test, with Benjamini-Hochberg false discovery rate (FDR) correction applied to adjust for multiple comparisons (adjusted *p*-value < 0.05 was considered significant). Linear Discriminant Analysis Effect Size (LEfSe) was subsequently performed using a logarithmic LDA score threshold of 2.0. Furthermore, a Random Forest ensemble learning model was constructed using the “RandomForest” package in R (version 4.2.1). The discriminative efficacy of bacterial species was assessed based on the Mean Decrease in Accuracy. To mitigate overfitting and evaluate model stability, 5-fold stratified cross-validation was performed and the out-of-bag (OOB) score was calculated. Candidate microbial markers were identified by intersecting the high-importance features from the Random Forest model with the significant taxa identified by LEfSe.

Statistical analyses and Receiver Operating Characteristic (ROC) curve analysis were performed using SPSS Version 23.0 (IBM, Armonk, NY, USA) and GraphPad Prism (version 9.0). The DeLong test was employed to compare Area Under the ROC curve (AUC) among candidate microbial markers and determine whether differences in discriminative performance were statistically significant. Post hoc power analysis was conducted to evaluate the statistical power of the observed AUC. Statistical significance was set at *p*-value < 0.05.

### 2.2. In Vitro Biofilm Co-Culture Model

#### 2.2.1. Bacterial Strains and Culture Conditions

The oral bacterial strains employed in this study were *Leptotrichia hofstadii* (GDMCC 1.4136, JCM 16775) and *Streptococcus mutans* (UA159). Columbia broth (BD Bacto, BD Biosciences, Franklin Lakes, NJ, USA) was used as the basal medium throughout the study to ensure consistency and comparability of experimental conditions. All cultures were maintained at 37 °C under strict anaerobic conditions (80% N_2_, 10% CO_2_, and 10% H_2_).

#### 2.2.2. Co-Cultured Biofilm Construction

Biofilms were performed in 24-well plates (Corning, Corning, NY, USA) under two nutritional conditions: a sucrose-free condition and a condition supplemented with 1% (*w*/*v*) sucrose. The initial optical densities (OD_600_) were adjusted to 0.05 for *S. mutans* and 0.10 for *L. hofstadii* in both monoculture and co-culture groups. Following inoculation, the plates were statically incubated within an anaerobic workstation. Biofilm samples were collected at specific time intervals (3, 6, 12, and 24 h) for downstream analyses.

#### 2.2.3. Biofilm Crystal Violet Staining

At each time point, the culture supernatant was aspirated, and plates were carefully rinsed by immersion in water to remove non-adherent bacteria [14]. Biofilms were stained with 500 μL of 0.1% (*w*/*v*) crystal violet (Solarbio, Beijing, China) for 30 min at room temperature. Following the removal of excess dye and subsequent washing, the bound stain was then eluted with 1 mL of 95% ethanol (Sinopharm, Beijing, China) for 15 min [15]. The biomass was then quantified by measuring the absorbance at 575 nm (OD_575_) using a microplate reader.

#### 2.2.4. Transwell Co-Culture System

To evaluate the effects of metabolites on biofilm formation, an indirect contact model was established using Transwell co-culture chambers (0.4 µm pore size, Corning, Corning, NY, USA) in 24-well plates. The initial inoculation concentrations were consistent with those used in the biofilm construction experiments. *L. hofstadii* and *S. mutans* were inoculated into the upper and lower chambers, respectively, with sterile Columbia broth in the upper chamber as the control group. At each designated time interval (3, 6, 12, and 24 h), the biofilms in the lower chambers were quantified by the crystal violet staining assay.

#### 2.2.5. Scanning Electron Microscopy (SEM)

To visualize biofilm architecture, sterile coverslips were placed at the bottom of 24-well plates prior to inoculation. At each designated time point (3, 6, 12, and 24 h), the coverslips were retrieved and gently rinsed with phosphate-buffered saline (PBS). The samples were then fixed with 3% (*v*/*v*) glutaraldehyde at 4 °C overnight. Following gradient dehydration using a series of ethanol concentrations, samples were subjected to critical point drying and gold sputter-coating. Specimens were observed using a scanning electron microscope (SEM; SU8020, Hitachi, Tokyo, Japan). For each sample, three fields were randomly selected at 1000× and 10,000× magnification.

#### 2.2.6. Confocal Laser Scanning Microscopy (CLSM)

Biofilms were established in 8-well chambered coverslips (Corning) for three-dimensional structural analysis. To visualize the spatial distribution of biofilm components, the samples were fluorescently labeled with SYTO™ 9 (Invitrogen, Carlsbad, CA, USA) to target bacterial nucleic acids and Alexa Fluor™ 647-labeled dextran (Invitrogen, Carlsbad, CA, USA; excitation/emission: 650/668 nm) to target the extracellular polysaccharide (EPS) [16].

The labeled biofilms were imaged using a confocal laser scanning microscope (Nikon AXR, Tokyo, Japan) equipped with a 40× water immersion objective. Z-stack scanning was performed to capture the full thickness and three-dimensional architecture of the biofilms. For each sample, three representative fields of view were randomly selected for acquisition. Subsequent image processing, including quantitative three-dimensional reconstruction and volumetric analysis of the biofilms, was conducted using Imaris software (version 10.1.0, Oxford Instruments, Abingdon, UK).

#### 2.2.7. pH Measurement

The pH of monoculture and co-culture supernatants was monitored in sterile tubes. At designated time intervals (0.5 h), the samples were centrifuged at 10,000 rpm for 10 min at 4 °C. The supernatant pH was measured with a precision pH meter (Mettler Toledo, Greifensee, Switzerland). The pH dynamics were subsequently constructed by plotting pH values against incubation time.

#### 2.2.8. Dual-Species Transcriptomics Sequencing

Biofilms formed by monocultures and co-cultures at 3 h were subjected to dual-species transcriptomics sequencing. The biofilms were collected and centrifuged at 10,000 rpm for 10 min at 4 °C. After the supernatant removal, the pellets were snap-frozen in liquid nitrogen and stored at −80 °C. Total RNA extraction and subsequent sequencing were performed by Majorbio Bio-pharm Technology Co., Ltd. (Shanghai, China). Libraries were prepared using the Illumina^®^ Stranded mRNA Prep Ligation Kit (San Diego, CA, USA). Sequencing was conducted on the Illumina NovaSeq 6000 platform. The raw reads were quality-filtered using fastp (version 0.23.4) and aligned to the reference genomes of *S. mutans* (UA159) and *L. hofstadii* using STAR (version 2.7.11b). Differential expression analysis was performed using DESeq2 (version 1.42.0), with significance thresholds set at a false discovery rate (FDR) < 0.05 and |log_2_FC| > 1. Functional annotation and metabolic pathway enrichment analysis was conducted using the Kyoto Encyclopedia of Genes and Genomes (KEGG) database.

#### 2.2.9. Data Processing and Statistical Analysis

Statistical analyses were performed using GraphPad Prism (version 9.0; GraphPad Software, San Diego, CA, USA). All statistical tests were two-tailed, and differences were considered statistically significant at *p* < 0.05. For comparisons between two experimental groups, the Wilcoxon rank-sum test was employed.

For imaging data analysis, three-dimensional reconstruction and surface rendering of CLSM images were conducted using Imaris (version 10.1.0; Oxford Instruments, UK). Quantitative parameters, including biofilm thickness, EPS volume, and bacterial volume, were calculated using the software’s integrated surface and measurement modules. All quantitative data are presented as the mean ± standard deviation (SD) from three independent biological replicates.

## 3. Results

### 3.1. Demographic Information and Clinical Characteristics of the Cohort

Supragingival plaque samples were collected one month after dental rehabilitation from 32 children with S-ECC. The cohort was divided into a sequencing set (n = 16) and a validation set (n = 16) based on the chronological order of enrollment. Clinical characteristics are summarized in Table S1.

The demographic and clinical characteristics of the sequencing set are summarized in Table 1. Comparative analysis using Fisher’s exact test and the Wilcoxon rank-sum test revealed no statistically significant differences between the non-recurrence and recurrence groups regarding sex (*p* = 1.000), age at initial visit (*p* = 0.878), baseline dmft index (*p* = 0.878), number of preformed metal crowns (PMCs) (*p* = 0.328), or number of remaining teeth (primary teeth remaining after dental rehabilitation) (*p* = 0.721). The demographic and clinical characteristics of the validation set are summarized in Table S2, and no statistically significant differences were observed between the groups for the same variables.

### 3.2. Supragingival Microbial Profile Differences Between Recurrence and Non-Recurrence Group

#### 3.2.1. Microbial Diversity and Taxonomic Composition Post-Treatment

Metagenomic sequencing of the 16 samples in the sequencing set yielded 73,343,934 high-quality reads, with an average read length of 622 bp and a non-redundant gene catalog consisting of 2753 sequences. The Q20 and Q30 scores for sequencing quality evaluation exceeded 94% across all samples. Taxonomic profiling via MetaPhlAn3 revealed that Bacteria were the predominant domain, accounting for 97.38% of the relative abundance, followed by minor fractions of Eukaryotes and Viruses. The identified bacterial taxa spanned 6 phyla, 11 classes, 23 orders, 32 families, 66 genera, and 240 species.

To elucidate the microbial profile at one month post-operatively, alpha and beta diversity were assessed (Figure 1B). While microbial richness (evaluated by the Chao index) showed no significant difference between the two groups (*p* = 0.4309), both the microbial diversity (evaluated by the Shannon index, *p* = 0.01008) and evenness (evaluated by the Shannoneven index, *p* = 0.007406) were significantly lower in the recurrence group compared with the non-recurrence group. In contrast, beta diversity analysis via PCoA revealed no clear separation in overall community structure between the two groups (Figure 1C; *p* = 0.145). The lower diversity and evenness in the recurrence group reflect possible microbial dysbiosis.

Taxonomic analysis at the genus level showed that both groups were dominated by core oral taxa, including *Corynebacterium*, *Actinomyces*, *Neisseria*, *Lautropia*, and *Streptococcus*, with no statistically significant differences in relative abundance (Figure 1D). Further species-level profiling of the top 20 dominant taxa, such as *Corynebacterium matruchotii*, *Lautropia mirabilis*, and *Actinobaculum* sp. HMT-183 similarly revealed a consistent distribution between the recurrence and non-recurrence groups (Figure 1E). While the dominant microbial composition was comparable between the two groups, specific low-abundance species were differentially enriched in each group. Species-level analysis was performed using the Wilcoxon rank-sum test, which identified seven differentially abundant taxa between the two groups (Figure S1).

#### 3.2.2. Identification of *Leptotrichia hofstadii* as a Recurrence-Associated Microbial Marker

**Figure 2:**
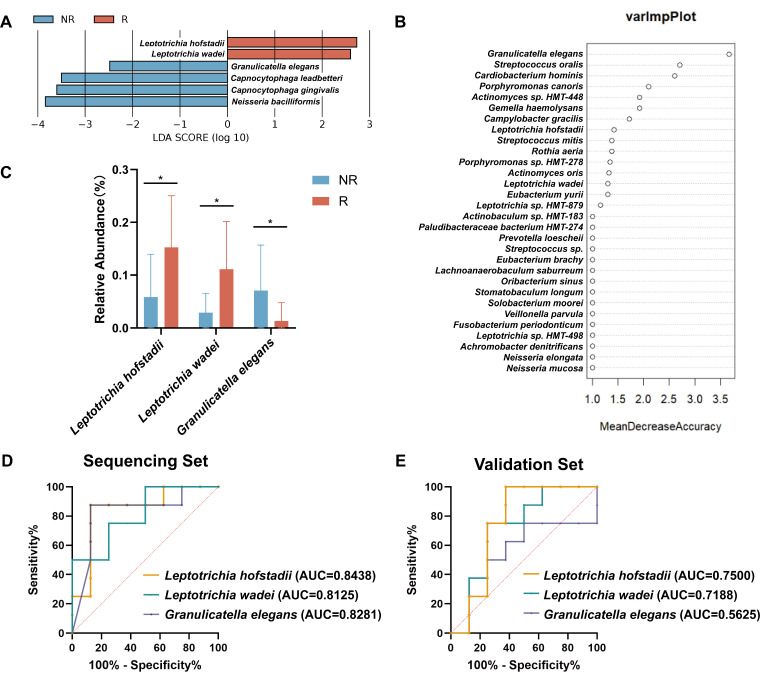
Screening and validation of microbial biomarkers associated with S-ECC recurrence (n = 8 per group in both sequencing and validation sets). (**A**) Linear discriminant analysis (LDA) effect size (LEfSe) analysis identifying differentially abundant species between non-recurrence (n = 8) and recurrence (n = 8) groups (LDA score > 2.0). (**B**) Random forest analysis showing the top 30 species ranked by mean decrease in accuracy. (**C**) Relative abundance comparison of the three overlapping biomarkers between groups. * *p*-value < 0.05 (**D**,**E**) Receiver operating characteristic (ROC) curves of candidate biomarkers in the sequencing set (**D**) and validation set (**E**).

To identify species-level biomarkers with significant discriminative power between the recurrence and non-recurrence groups, LEfSe analysis was performed (LDA score > 2.0). *Leptotrichia hofstadii* and *Leptotrichia wadei* were significantly enriched in the recurrence group, while *Granulicatella elegans*, *Capnocytophaga leadbetteri*, *Capnocytophaga gingivalis*, and *Neisseria bacilliformis* were associated with the non-recurrence group (Figure 2A). Random Forest model was subsequently employed to rank the contribution of each species to group classification based on permutation importance (Mean Decrease in Accuracy). Among the top 30 species identified by the model, *L. hofstadii*, *L. wadei*, and *G. elegans* overlapped with the LEfSe differential analysis results (Figure 2B). Between-group comparisons using the Wilcoxon rank-sum test further confirmed that the relative abundances of these three taxa differed significantly between the two groups (Figure 2C).

The diagnostic efficacy of these three candidate markers was evaluated using Receiver Operating Characteristic (ROC) analysis. In the sequencing set, all three species demonstrated favorable discriminative performance with area under the curve (AUC) values exceeding 0.8. The AUC values for *L. hofstadii, L. wadei* and *G. elegans* were 0.8438, 0.8125 and 0.8281, respectively (Figure 2D). However, in the independent validation set, *L. hofstadii* maintained the highest predictive value with an AUC of 0.7500 (Figure 2E), whereas the performance of *L. wadei* (AUC = 0.7188) and *G. elegans* (AUC = 0.5625) was comparatively lower. Consequently, *L. hofstadii* was identified as the most consistent microbial marker associated with S-ECC recurrence and was selected as the target organism for subsequent in vitro mechanistic investigations.

To evaluate Random Forest model stability, 5-fold stratified cross-validation was performed, yielding an AUC of 0.900 ± 0.200 in the sequencing set, with an out-of-bag (OOB) score of 0.562. These results indicate that the training estimates were overly optimistic. In the sequencing set, the AUC for *L. hofstadii* was 0.844, 95% confidence interval (CI) [0.583–1.000]; for *L. wadei*, AUC was 0.812, 95% CI [0.539–0.984]; and for *G. elegans*, AUC was 0.828, 95% CI [0.587–1.000]. In the validation set, *L. hofstadii* showed an AUC of 0.750, 95% CI [0.467–1.000]; *L. wadei* showed an AUC of 0.719, 95% CI [0.406–0.969]; and *G. elegans* showed an AUC of 0.563, 95% CI [0.250–0.857]. Wide confidence intervals in both datasets reflected high uncertainty under small-sample conditions. Post hoc power analysis for *L. hofstadii* (Cohen’s d = 1.05) indicated an achieved power of approximately 0.50. DeLong’s test for pairwise comparison of ROC curves revealed no significant difference between *L. hofstadii* and *L. wadei* (sequencing set: *p* = 0.748; validation set: *p* = 0.142), with *G. elegans* exhibiting inconsistent discriminative direction between the two datasets. Despite these limitations, multiple validation methods support the authenticity of the observed discriminative signals rather than their arising from entirely random noise. Stratified permutation tests demonstrated that in the sequencing set, *L. hofstadii* (*p*-value = 0.010) and *L. wadei* (*p*-value = 0.014) were unlikely to arise by chance. In the validation set, *L. hofstadii* maintained a consistent direction of discrimination, with a permutation *p*-value of 0.052 marginally exceeding the 0.05 threshold. Its effect size (Cohen’s d ≈ 0.9) was comparable to that in the sequencing set (Cohen’s d = 1.05), supporting the authenticity of the signal rather than random fluctuation. *L. wadei* showed a permutation test *p*-value of 0.082, while *G. elegans* showed a *p*-value of 0.359, failing to reach significance. *L. hofstadii* given its most stable direction across both datasets, can be prioritized as a candidate marker for further validation in larger samples.

### 3.3. Synergistic Interactions Between L. hofstadii and S. mutans in Co-Culture Biofilms

#### 3.3.1. *L. hofstadii* Promotes Early Biofilm Formation in Co-Culture

Although *Leptotrichia hofstadii* is capable of producing acid through carbohydrate metabolism, its contribution to caries development has often been questioned due to its limited acid tolerance and relatively slow growth compared with dominant supragingival taxa such as *Streptococcus* [17]. Therefore, we hypothesized that *L. hofstadii* might exert its influence through synergistic interactions with key cariogenic species. To test this hypothesis, we established a co-culture model with *Streptococcus mutans*.

**Figure 3:**
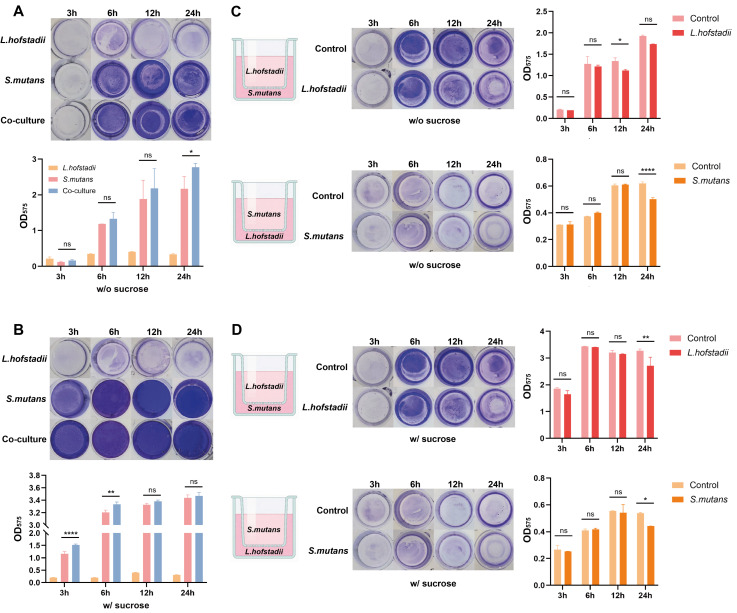
Biofilm formation of *L. hofstadii* and *S. mutans* under direct and indirect contact co-culture conditions (n = 3 biological replicates per group). (**A**,**B**) Crystal violet staining and quantification of biofilm biomass in monoculture and direct contact co-culture without sucrose (**A**) and with 1% sucrose (**B**). (**C**,**D**) Transwell co-culture assays evaluating the effect of indirect contact on biofilm formation without sucrose (**C**) and with 1% sucrose (**D**); crystal violet staining and quantification of biofilm biomass were performed in the lower chamber. ns: not significant, * *p*-value < 0.05, ** *p*-value < 0.01, **** *p*-value < 0.0001.

Under sucrose-free conditions, total co-culture biomass significantly exceeded that of either monoculture only at the 24 h time point (Figure 3A). In contrast, the presence of 1% sucrose induced sucrose-dependent enhancement of biofilm formation in *S. mutans*, with significantly higher biomass at all time points compared with the sucrose-free groups. Notably, the co-culture system demonstrated a synergistic effect during the early stages of development (3 and 6 h). At 3 h, the co-culture biomass was significantly higher than that of the *S. mutans* monoculture, a trend that persisted at 6 h (Figure 3B). As incubation progressed to 12 and 24 h, the biomass of the co-culture and *S. mutans* monoculture showed no statistically significant differences.

To determine whether the observed synergy was mediated by secreted factors, a Transwell co-culture system was employed. This model allows for free exchange of medium and metabolic products while physically separating the two species. We assessed the reciprocal dynamic effects between *L. hofstadii* and *S. mutans* under both sucrose-free and sucrose-supplemented conditions.

In the absence of sucrose, no significant mutual promotion was observed between *L. hofstadii* and *S. mutans* (Figure 3C). At 24 h, co-culture biofilm biomass was significantly lower than the control. Similarly, in the presence of 1% sucrose, the Transwell co-culture system failed to replicate the synergistic promotion seen in direct contact models (Figure 3D). Between 3–12 h, no significant differences were observed compared with monoculture controls. However, with prolonged incubation, this interaction shifted to an inhibitory effect. These results show that the cooperative interaction between *L. hofstadii* and *S. mutans* is dependent on direct spatial interactions rather than diffusible metabolic mediators.

**Figure 4:**
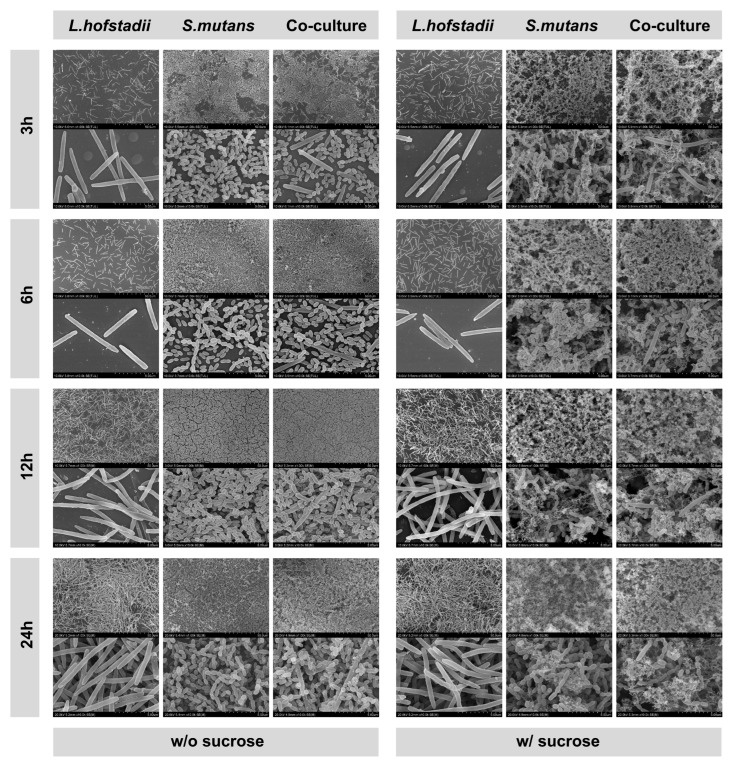
Scanning electron microscopy (SEM) images of biofilms formed by monoculture and co-culture of *L. hofstadii* and *S. mutans* (n = 3 biological replicates per group). Biofilms were cultured without (left panel) or with 1% (*w*/*v*) sucrose (right panel) for 3, 6, 12, and 24 h. Upper rows show low-magnification views (1000×), and lower rows show high-magnification views (10,000×) of representative areas (three fields of view randomly selected per sample).

To further elucidate how these interspecies interactions modulate biofilm architecture at the ultrastructural level, scanning electron microscopy (SEM) was employed (Figure 4). At 1000× magnification, observation revealed that in the presence of 1% sucrose, *S. mutans* produced an abundance of filamentous extracellular polymeric substances (EPS) both in the monoculture and co-culture compared with the absence of sucrose. High-magnification (10,000×) images provided insights into the spatial organization of the dual-species community: *L. hofstadii* bacterial cells were observed interweaving among *S. mutans*, functioning as a spatial scaffold that created a loose, porous architecture characterized by numerous microchannels. Interestingly, at 24 h, fewer *L. hofstadii* units were visible on the biofilm surface, suggesting they may have been encapsulated within the deep layers of the matrix formed by *S. mutans* and its dense EPS.

#### 3.3.2. Co-Culture Enhances Early Biofilm Formation and EPS Production, and Accelerates Environmental Acidification

The preceding experiments revealed that under 1% (*w*/*v*) sucrose conditions, the co-culture system exhibits a significant synergistic effect during the early stage of biofilm formation. To further quantify the biofilm biomass and EPS formation at 3 and 6 h of incubation, CLSM was used to image the monoculture and co-culture in the presence of 1% (*w*/*v*) sucrose.

**Figure 5:**
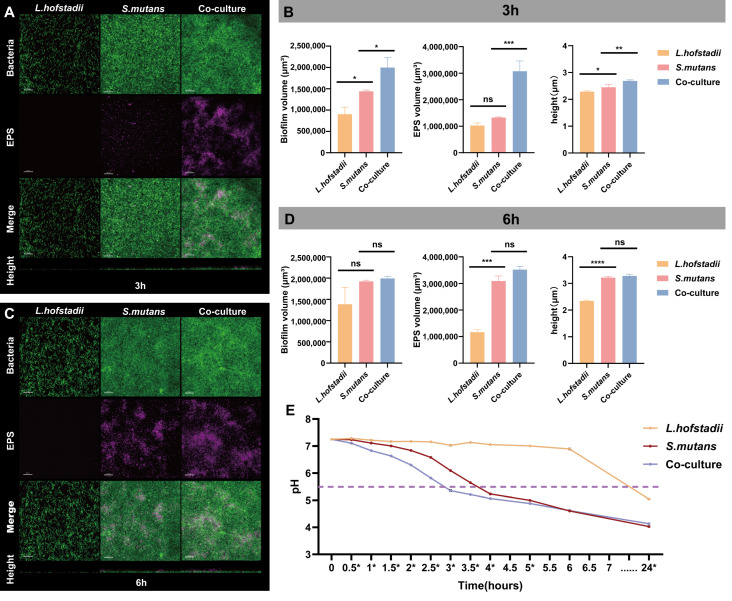
Confocal laser scanning microscopy (CLSM) analysis of extracellular polysaccharide (EPS) production and pH dynamics (n = 3 biological replicates per group). (**A**,**C**) CLSM images of biofilms formed by *L. hofstadii*, *S. mutans*, and their co-culture at 3 h (**A**) and 6 h (**C**), stained with SYTO 9 (green, bacteria) and Alexa Fluor 647-conjugated dextran (far-red, EPS). Scale bars, 50 μm. (**B**,**D**) Quantitative analysis of biofilm volume, EPS volume, and biofilm height at 3 h (**B**) and 6 h (**D**). (**E**) pH dynamics of culture supernatants over 24 h. The *p*-values shown on the x-axis represent the results of pairwise comparisons between the *S. mutans* monoculture and co-culture groups. ns: not significant, * *p*-value < 0.05, ** *p*-value < 0.01, *** *p*-value < 0.001, **** *p*-value < 0.0001.

CLSM at 3 h (Figure 5A) showed that *L. hofstadii* monocultures produced a trace amount of EPS fluorescence, and the *S. mutans* monoculture exhibited scattered EPS signals. In contrast, the co-culture displayed a marked EPS signal. Quantitative analysis (Figure 5B) confirmed that both biofilm volume and EPS volume in the co-culture were significantly higher than those in the *S. mutans* monoculture, accompanied by a significant increase in overall biofilm height. The merged images revealed a high degree of spatial overlap between the far-red EPS fluorescence and green bacterial signals, indicating that *L. hofstadii* substantially facilitates the early recruitment of *S. mutans* and enhances the amount of EPS.

By 6 h, while *L. hofstadii* continued to exhibit weak biofilm-forming capacity, and the *S. mutans* monoculture showed an increase in biofilm volume and EPS signals (Figure 5C). At this stage, the co-culture showed no statistically significant differences in bacterial biomass or EPS levels compared with *S. mutans* monocultures (Figure 5D). These findings suggest that the stimulatory effect of the interspecies interaction on biofilm accumulation is most pronounced during the early phase.

The pH dynamic curve (Figure 5E) presented the pH values at each time point for *S. mutans* and *L. hofstadii* monocultures and co-culture. *L. hofstadii* monocultures maintained a nearly neutral environment (pH ≈ 7.0) during the first 6 h, showing minimal acidogenic activity. In contrast, *S. mutans* monocultures exhibited a progressive pH decline, reaching the critical enamel demineralization threshold (pH 5.5) between 3.5 and 4 h. Notably, the co-culture group demonstrated a significantly accelerated acidification rate, with the pH dropping below 5.5 within the first 2.5–3 h. Although pH values in the two groups eventually converged during the later stages (6–24 h), the co-culture reached a cariogenic pH environment significantly earlier. These results show that *L. hofstadii* accelerates the attainment of a critical acidic microenvironment when co-cultured with *S. mutans*.

#### 3.3.3. *L. hofstadii* Enhances the Expression of Carbohydrate Metabolism and Signal Transduction Genes in Co-Culture

To investigate the transcriptional differences during the early stage of biofilm formation (3 h), dual-species transcriptomics sequencing was performed. We specifically focused on the expression profiles of virulence factors associated with biofilm formation and metabolic activity. Hierarchical clustering revealed distinct transcriptional signatures for both *S. mutans* and *L. hofstadii* under co-culture conditions compared with their respective monocultures.

A total of nine samples were sequenced, comprising three biological replicates per group. The clean reads per sample ranged from 41,816,276 to 55,174,696 (mean, 46,500,000), yielding clean bases ranging from 6.19 Gb to 8.23 Gb (mean, 6.9 Gb). The GC content ranged from 35.86% to 40.77% (mean, 39.5%), consistent with typical oral microbiome composition. The proportion of high-quality clean reads relative to raw reads ranged from 97.49% to 99.56% (mean, 98.5%), indicating satisfactory sequencing data quality for downstream bioinformatics analyses.

**Figure 6:**
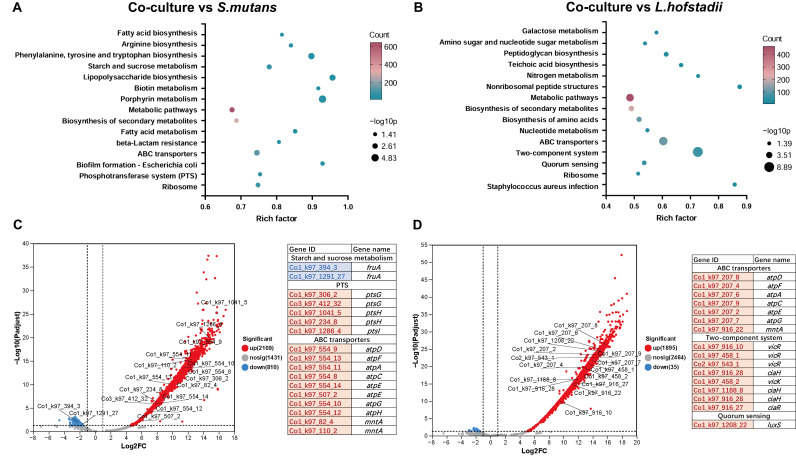
Dual-species transcriptomics analysis of biofilms formed by *L. hofstadii* and *S. mutans* under monoculture and co-culture conditions (n = 3 biological replicates per group). (**A**) KEGG pathway enrichment analysis of upregulated genes in co-culture compared with *S. mutans* monoculture. (**B**) KEGG pathway enrichment analysis of upregulated genes in co-culture compared with *L. hofstadii* monoculture. (**C**) Volcano plot showing differentially expressed genes between co-culture and *S. mutans* monoculture. Key genes associated with starch and sucrose metabolism, phosphotransferase system (PTS), and ABC transporters are highlighted in the table. (**D**) Volcano plot showing differentially expressed genes between co-culture and *L. hofstadii* monoculture. Key genes associated with ABC transporters, two-component system, and quorum sensing are highlighted in the inset table.

KEGG pathway enrichment analysis was conducted on the differentially expressed genes (DEGs) between the co-culture and the *S. mutans* monoculture. The enrichment bubble plot (Figure 6A) revealed that DEGs were significantly enriched in multiple pathways, including starch and sucrose metabolism and the phosphotransferase system (PTS). Activation of the PTS and sucrose metabolism pathways suggests that co-culturing with *L. hofstadii* may enhance the sugar uptake efficiency of *S. mutans*. The volcano plot (Figure 6C) revealed downregulation of *fruA* (fructanase), while key PTS components, including *ptsG* (glucose-specific phosphotransferase system), *ptsH* (phosphocarrier protein HPr), and *ptsI* (PEP-protein phosphotransferase), were significantly upregulated.

Transcriptomic analysis in *L. hofstadii* within the co-culture revealed significant upregulation of ABC transporter components, particularly ATP synthase subunits (*atpA/C/D/E/F/G*) and the manganese transport protein *mntA* (Figure 6D). Furthermore, core regulatory genes associated with two-component systems (TCS) and quorum sensing, including *ciaR/H* (carbon metabolism regulation) and *vicR/K* (cell wall metabolism and virulence regulation), as well as *luxS*, were significantly upregulated.

## 4. Discussion

The oral microenvironment of the recurrence group may have been biologically primed for future relapse during the initial phase of recolonization, well before clinical manifestations. The differences in supragingival plaque species composition observed at one month after treatment in our study are consistent with a previous investigation [9], collectively supporting this finding. Although no significant differences were observed in high abundance species between the two groups (Figure 1E), certain low abundance species like *Leptotrichia hofstadii*, *Leptotrichia* sp. HMT-879 and *Leptotrichia wadei* appeared to contribute to S-ECC recurrence (Figure S1).

Previous studies have demonstrated that comprehensive treatment under general anesthesia significantly reduces the microbial load of *S. mutans* through mechanical debridement and antimicrobial interventions [13], with suppression persisting for at least three months post-operatively [18]. Notably, at the one-month follow-up in our study, no statistically significant difference was observed between the two groups regarding the relative abundance of *S. mutans* (Figure S2). This suggests that *S. mutans* may not serve as the predominant driver during the early stages of microbial re-establishment. However, temporal dynamics appear to shift as the disease progresses. Tanner A C et al. reported a higher detection rate of *S. mutans* in the recurrence group at the time of clinical recurrence compared with non-recurrence follow-ups [19]. Furthermore, previous research has shown that while the relative abundance of *S. mutans* on healthy surfaces in the non-recurrence group is significantly higher than that on healthy surfaces in the recurrence group, its abundance within the active carious lesions of the recurrence group remains substantially higher than in all other assessed sites [11]. These observations collectively imply that while *S. mutans* remains the predominant cariogenic pathogen within the carious niche, the progression toward recurrence is likely facilitated by other species. These taxa may construct a synergistic microenvironment that does not acutely increase *S. mutans* abundance but instead promotes its virulence expression and ecological dominance during the subsequent stages of caries.

Our in vitro experiments also provide partial support for this perspective. The contact-dependent synergy between *L. hofstadii* and *S. mutans* observed in our experiments provides preliminary support for an “indirect driving” hypothesis. Previous metagenomic studies on primary S-ECC have identified genera such as *Rothia* and *Lactobacillus* [20,21], whereas the specific role of *Leptotrichia* has remained largely underexplored.

Our in vitro investigations revealed that *S. mutans* and *L. hofstadii* co-culture resulted in increased total biomass, enhanced EPS formation, and an accelerated acidification rate, which caused the local environment to reach the critical demineralization threshold (pH 5.5) significantly earlier. Notably, this interaction exhibits a distinct time-dependent pattern, manifesting predominantly during the early stages of biofilm formation (3 h). This early-stage advantage is critical, as initial colonization efficiency dictates the subsequent structural stability and cariogenic potential of the mature biofilm [22,23]. We propose three complementary mechanisms for this synergy. First, metabolic enhancement: transcriptomic data revealed the upregulation of *S. mutans* PTS genes (*ptsG/H/I*) and ABC transporters (*atp* family), suggesting heightened sugar uptake and energy metabolism efficiency. Second, physical scaffolding: SEM imaging demonstrated that the elongated *L. hofstadii* units interdigitate with *S. mutans*, forming a porous “spatial scaffold” that may potentially facilitate nutrient diffusion and localized acid accumulation. Third, interspecies signaling: the significant upregulation of *L. hofstadii luxS* suggests that AI-2-mediated quorum sensing may possibly coordinate the collective virulence behaviors of the dual-species community [24,25].

In our study, CLSM imaging revealed a significant increase in EPS production within the co-culture biofilm, which aligns closely with our observation of the remarkable downregulation of *fruA*. Mechanistically, the primary function of FruA involves the degradation of fructan rather than glucan synthesis. Thus, its reduced expression could directly reduce the catabolic flux of EPS, thereby facilitating net accumulation [26]. Furthermore, evidence suggests that FruA hydrolyzes sucrose prior to the secretion of GTFs and subsequent glucan synthesis, and the glucose derived from this hydrolysis is not utilized for glucan construction [27]. Consequently, the downregulation of *fruA* observed in our study not only obstructs the degradation pathway of EPS but may also mitigate the unproductive consumption of synthetic substrate. Notably, the transcription levels of *gtf*, which are the key genetic drivers promoting sucrose-dependent adherence and robust acidogenesis in *S. mutans* [28,29], showed no detectable upregulation in this study. While this phenomenon appears seemingly contradictory to the observed increase in EPS, when considered with the expression profile of *fruA*, it implies that the virulence of *S. mutans* within co-culture biofilms might be subject to complex post-transcriptional and post-translational regulations [30,31].

Despite these findings, several limitations should be acknowledged. The relatively small sample size substantially constrained the statistical power for biomarker screening, as supported by limited generalizability of the Random Forest model, the wide confidence intervals of ROC curves, and the modest post hoc power analysis results, all of which collectively indicate that the current candidate biomarkers are insufficient for robust clinical application. Further research is warranted to incorporate multi-dimensional clinical and microbiological parameters with expanded sample sizes to establish a robust predictive framework. Therefore, the primary objective of this study was to explore microbial indicators associated with S-ECC recurrence and to preliminarily investigate potential cariogenic mechanisms in vitro, rather than to construct a precise predictive model. Additionally, it should be noted that *L. hofstadii* is not the only microbial species associated with S-ECC recurrence. *L. wadei* exhibited comparable discriminative performance in our analysis, and its potential cariogenic role warrants further investigation. Another limitation lies in the absence of in vivo validation, which restricts our ability to confirm causal relationships between the identified microbial biomarkers and caries recurrence. Further studies utilizing animal models or longitudinal clinical cohorts are warranted to address this gap.

## 5. Conclusions

This study identified *L. hofstadii* as a candidate microbial biomarker for predicting S-ECC recurrence at one month post-operative using the metagenomic sequencing method. Further in vitro dual-species experiments revealed that *L. hofstadii* promotes early-stage *S. mutans* colonization and EPS formation through contact-dependent interactions. In addition, this synergy significantly accelerates environmental acidification, reaching the demineralization threshold of pH 5.5 earlier than *S. mutans* monocultures. This process is accompanied by the transcriptional upregulation of carbohydrate metabolism pathways, such as the PTS and ABC transporters, and the downregulation of *fruA* gene expression, which could inhibit EPS degradation. Ultimately, this synergistic co-culture model offers a novel perspective for post-operative monitoring and the development of precision prevention strategies targeting early biofilm assembly to mitigate caries recurrence.

## Figures and Tables

**Table 1:** Comparison of Baseline Characteristics in the Sequencing Set.

	Non-Recurrence	Recurrence	*p*-Value
Sample size	n = 8	n = 8	
Sex (Male/Female) ^a^	4/4	3/5	1.000
Age at initial visit ^b^ (median, IQR)	3.9(3.2–4.8)	3.6(3.4–4.2)	0.833
dmft (median, IQR)^b^	16(14–18)	16(13–19)	0.831
Number of PMCs ^1,b^ (median, IQR)	4(0–4)	4(3–5)	0.270
Remaining teeth (median, IQR) ^b^	20(20–20)	20(20–20)	0.317
Time of recurrence (months, mean ± SD)		12.0 ± 7.1	

^1^ PMCs: Preformed Metal Crowns. ^a^ Fisher’s exact test. ^b^ Wilcoxon rank-sum test.

## Data Availability

The original contributions presented in this study are included in the article/Supplementary Material. Further inquiries can be directed to the corresponding authors.

## Supplementary Materials

The following supporting information can be downloaded at: https://www.mdpi.com/article/10.3390/microorganisms14071513/s1, Table S1: Demographic and Clinical Characteristics of Enrolled Children. Table S2: Comparison of Baseline Characteristics in the Validation Set. Figure S1: Comparative analysis of the relative abundance of significantly differentially abundant species between the non-recurrence group and recurrence group. Statistical significance was determined by the Wilcoxon rank-sum test. All species shown met the significance threshold of *p*-value < 0.05. Figure S2: Comparative analysis of the relative abundance of *Streptococcus mutans* one month post-operatively. (A) Sequencing set. (B) Validation set. (C) Individual sample distribution across both sets. Statistical significance was determined by the Mann–Whitney U test. ns: not significant.

## Abbreviations

The following abbreviations are used in this manuscript:

S-ECC	Severe Early Childhood Caries
NR	Non-recurrence
R	Recurrence
EPS	Extracellular Polysaccharides
SEM	Scanning Electron Microscopy
CLSM	Confocal Laser Scanning Microscopy
OD	Optical Density
DEGs	Differentially Expressed Genes
PTS	Phosphotransferase System
TCS	Two-component System
GTFs	Glucosyltransferases
ROC	Receiver Operating Characteristic
AUC	Area Under the Curve
LEfSe	Linear Discriminant Analysis Effect Size
LDA	Linear Discriminant Analysis
