# Supplementary material for: Identification of Leptotrichia hofstadii as a Post-Treatment Recurrence Biomarker in Severe Early Childhood Caries
Source: Microorganisms. 2026 Jul 11;14(7):1513. doi: 10.3390/microorganisms14071513 (PMC13414078; doi:10.3390/microorganisms14071513)
Supplement: Supplementary file 1 [file microorganisms-14-01513-s001.zip › microorganisms-4371180-supplementary.pdf]

Supplementary Materials:

Table S1. Demographic and Clinical Characteristics of Enrolled Children.

Sample ID	Group	Characteristics at Initial Visit			Post-operative Characteristics	
		Sex	Age	dmft	Number of PMCs[1]	Remaining Teeth
**Sequencing Set**						
1	NR	Female	4.2	16	5	20
2	NR	Female	3.6	14	1	20
3	NR	Male	4.1	18	4	20
4	NR	Female	3.2	16	4	20
5	NR	Male	2.6	18	0	20
6	NR	Male	5.2	14	0	20
7	NR	Male	3.3	18	4	20
8	NR	Female	5.0	13	4	20
9	R	Female	4.3	15	5	17
10	R	Male	2.8	16	0	20
11	R	Female	3.7	14	6	20
12	R	Female	3.4	13	2	20
13	R	Male	3.4	13	4	20
14	R	Female	3.4	20	4	20
15	R	Male	4.8	16	4	20
16	R	Female	3.7	20	5	20
**Validation Set**						
1	NR	Male	4.2	12	4	20
2	NR	Female	4.3	15	5	19
3	NR	Female	3.3	12	3	20
4	NR	Male	2.9	14	4	20
5	NR	Male	2.8	16	4	20
6	NR	Male	3.6	15	3	19
7	NR	Female	4.3	11	1	20
8	NR	Male	3.6	18	4	20
9	R	Female	2.9	14	0	20
10	R	Female	3.1	20	4	20
11	R	Male	3.9	14	0	18
12	R	Female	3.3	15	0	20
13	R	Female	3.9	11	5	20
14	R	Male	3.9	14	0	20
15	R	Male	3.1	10	2	20
16	R	Male	4.3	11	2	20

[1] PMCs：Preformed Metal Crowns.

Table S2. Comparison of Baseline Characteristics in the Validation Set.

	Non-recurrence	Recurrence	p-value
**Sample size**	n=8	n=8	
**Sex** (Male/Female) [a]	5/3	4/4	1.000
**Age at initial visit** [b] (median, IQR)	3.6 (3.0-4.3)	3.6(3.1-3.9)	0.832
**dmft** (median, IQR)[b]	15(12-16)	14(11-15)	0.424
**Number of PMCs** [1, b] (median, IQR)	4(3-4)	1(0-4)	0.060
**Remaining teeth** (median, IQR) [b]	20(19-20)	20(20-20)	0.643
**Time of recurrence** (mean, SD)		9.1±3.7	

[1] PMCs：Preformed Metal Crowns.

[a] Fisher's exact test

[b] Wilcoxon rank-sum test

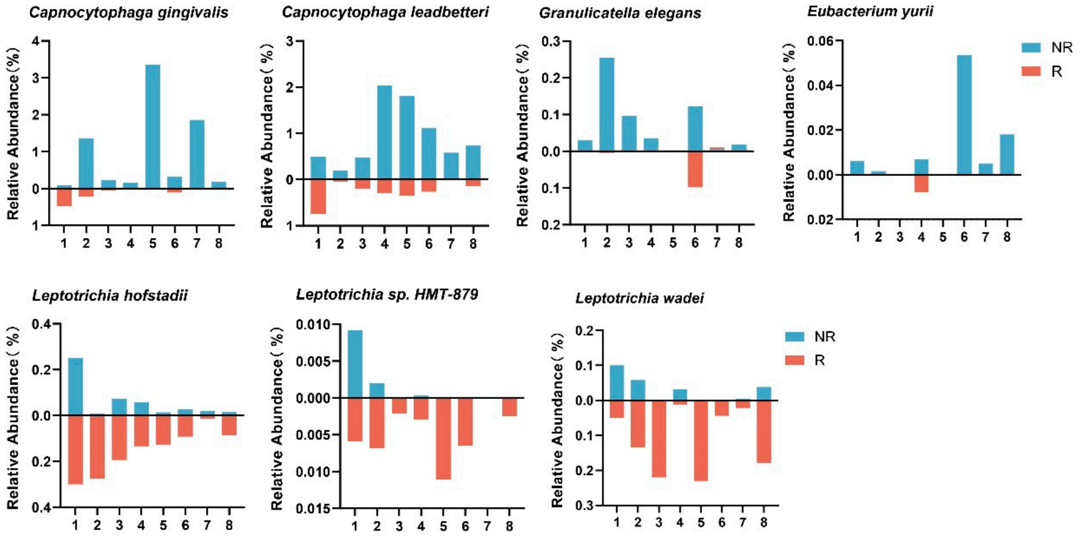

Figure S1. Comparative analysis of the relative abundance of significantly differentially abundant species between the non-recurrence group and recurrence group. Statistical significance was determined by the Wilcoxon rank-sum test. All species shown met the significance threshold of p-value < 0.05.

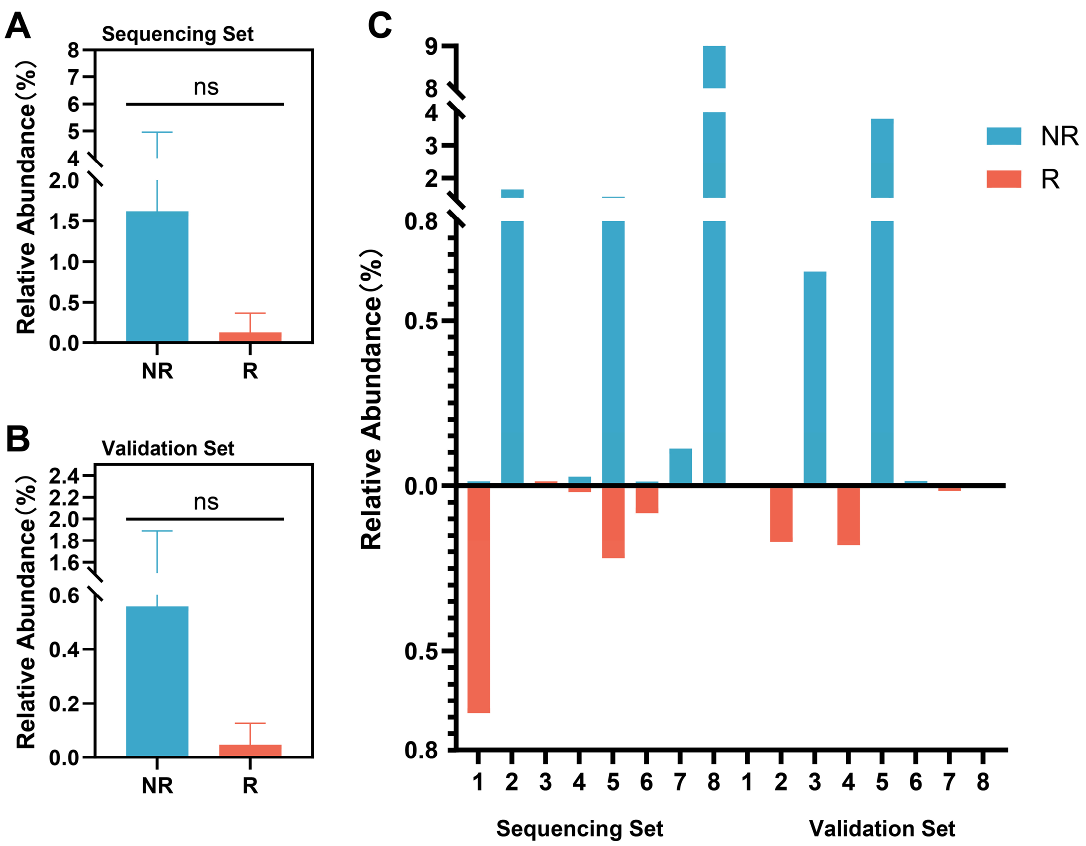

Figure S2. Comparative analysis of the relative abundance of *Streptococcus mutans* one month post-operatively. (A) Sequencing set. (B) Validation set. (C) Individual sample distribution across both sets. Statistical significance was determined by the Mann–Whitney U test. ns: not significant.
